# Estimating required information size by quantifying diversity in random-effects model meta-analyses

**DOI:** 10.1186/1471-2288-9-86

**Published:** 2009-12-30

**Authors:** Jørn Wetterslev, Kristian Thorlund, Jesper Brok, Christian Gluud

**Affiliations:** 1 Copenhagen Trial Unit, Centre for Clinical Intervention Research, Department 3344, Rigshospitalet, Copenhagen University Hospital, Blegdamsvej 9, DK-2100 Copenhagen Ø, Denmark

## Abstract

**Background:**

There is increasing awareness that meta-analyses require a sufficiently large information size to detect or reject an anticipated intervention effect. The required information size in a meta-analysis may be calculated from an anticipated *a priori *intervention effect or from an intervention effect suggested by trials with low-risk of bias.

**Methods:**

Information size calculations need to consider the total model variance in a meta-analysis to control type I and type II errors. Here, we derive an adjusting factor for the required information size under any random-effects model meta-analysis.

**Results:**

We devise a measure of diversity (*D*^2^) in a meta-analysis, which is the relative variance reduction when the meta-analysis model is changed from a random-effects into a fixed-effect model. *D*^2 ^is the percentage that the between-trial variability constitutes of the sum of the between-trial variability and a sampling error estimate considering the required information size. *D*^2 ^is different from the intuitively obvious adjusting factor based on the common quantification of heterogeneity, the inconsistency (*I*^2^), which may underestimate the required information size. Thus, *D*^2 ^and *I*^2 ^are compared and interpreted using several simulations and clinical examples. In addition we show mathematically that diversity is equal to or greater than inconsistency, that is *D*^2 ^≥ *I*^2^, for all meta-analyses.

**Conclusion:**

We conclude that *D*^2 ^seems a better alternative than *I*^2 ^to consider model variation in any random-effects meta-analysis despite the choice of the between trial variance estimator that constitutes the model. Furthermore, *D*^2 ^can readily adjust the required information size in any random-effects model meta-analysis.

## Background

Outcome measures in a single randomised trial or a meta-analysis of several randomised trials are typically dichotomous, especially for important clinical outcomes such as death, acute myocardial infarction, etc. Although meta-analysts cannot directly influence the number of participants in a meta-analysis like trialists conducting a single trial, the assessment of the meta-analytic result depends heavily on the amount of information provided. A limited number of events from a few small trials and the associated random error may be under-recognised sources of spurious findings. If a meta-analysis is conducted before reaching a required information size (i.e., the required number of participants in a meta-analysis) it should be evaluated according to the increased risk that the result may represent a chance finding. It has recently been suggested that sample size estimation in a single trial may be less important in the era of systematic review and meta-analysis [1]. Therefore, the reliability of a conclusion drawn from a meta-analysis, despite standardly calculated confidence limits, may depend even more on the number of events and the total number of participants included than hitherto perceived [2-8]. Both numbers determine the amount of available information in a meta-analysis. The information size (*IS*) required for a reliable and conclusive meta-analysis may be assumed to be at least as large as the sample size (*SS*) of a single well-powered randomised clinical trial to detect or reject an anticipated intervention effect [2-4].

The estimation of a required information size for a meta-analysis in order to detect or reject an anticipated intervention effect on a binary outcome measure should be considered based on reasonable assumptions. These assumptions may be derived from two kinds of information. Firstly, by anticipating an *a priori *intervention effect, most appropriately decided at the time when the protocol for a systematic review is prepared. An *a priori *intervention effect may be estimated by consulting related interventions for the same disease or the same intervention for related diseases suggesting a clinically relevant effect to be detected or ruled out [2-4]. This situation would be almost analogous to the hypothesis testing in a single randomised trial. Secondly, an intervention effect estimated by trials with low-risk of bias in the meta-analysis may represent our best estimate, at a given time point, of a possible intervention effect knowing the available data [5]. This would be a kind of a *post hoc *analysis of the information needed to detect or reject an intervention effect suggested by data already available. When planning a new trial it may be very important to estimate which *IS *is needed for the updated meta-analysis to be conclusive. In both instances the estimated required information size may be applied to grade the evidence reported in a cumulative meta-analysis adjusting for the risk of random error due to repetitive testing on accumulating data [5,6]. If the number of actually accrued participants falls short of the required *IS *the meta-analysis may be inconclusive even though the confidence interval is suggestive of a clinical relevant effect or. Because if the confidence interval (or the p-value) is appropriately adjusted with sequential methods, it may no longer show a statistically significant or clinically relevant effect. Conversely, if the actually accrued number of participants supersedes the required information size without the meta-analysis becoming statistically significant we may be able to rule out the anticipated intervention effect size [5].

It is not realistic to assume that the population of the included trials in a meta-analysis is truly homogenous, as it may be in a single clinical trial. Meta-analysis, therefore, should not analyse included participants as if they are coming from one trial [9]. Consequently the difference between obtaining the required *IS *and *SS *is rooted in the underlying assumption of between trial variability, and thus, the chosen meta-analytical model.

If the between-trial variability of the outcome measure estimates in a meta-analysis is incorporated into the model using the traditional one-way random-effects model, the required *IS *will be affected [5]. In this vein, the required *IS *is a monotonically increasing function of the total variability among the included trials. An estimate of the required *IS *can therefore be derived once the degree of variability is known or prespecified [5]. The test statistic for heterogeneity in a meta-analysis, the inconsistency factor (*I*^2^) based on Cochran's *Q *proposed by Higgins and Thompson [10], may seem an obvious quantity to use for this purpose as it allow us to estimate the degree of the variation, which is not covered by assumption of homogeneity [5]. However, *I*^2 ^is derived using a set of general assumptions that may be inappropriate in this context.

In this paper we derive a general expression for the required *IS *in any random-effects model. We prove the monotone relationship between *IS *and the degree of total variability in a one-way random-effects meta-analysis. We use our results to define a quantification of diversity (*D*^2^) between included trials in a meta-analysis, which is the relative model variance reduction when the model of pooling is changed from a random-effects model into a fixed-effect model. We analyse and discuss the differences between our definition of diversity, *D*^2^, and the commonly used measure for heterogeneity, *I*^2^.

## Methods

### 2.1 Deriving the required meta-analysis information size and diversity

If the required *IS *needed to detect or reject an intervention effect in a meta-analysis should be at least the sample size needed to detect or reject a similar effect in a single trial, then the following scenario applies:

Let *μ*_F _denote the weighted mean intervention effect to be detected in a fixed-effect model meta-analysis and let *μ*_R _denote the weighted mean intervention effect to be detected in a in a random-effects model meta-analysis using generic inverse variance weighting. The information size (*N*_F_) needed to reject an intervention effect *μ*_F _in the fixed-effect model (with a type I error less than *α*, a type II error less than *β*, and equal group sizes) becomes [11,12]:(2.1)

and the information size (*N*_R_) needed to reject *μ*_R _in the random-effects model (with a type I error less than *α*, a type II error less than *β*, and equal group sizes) becomes [11,12]:(2.2)

Where  and  are the variances in the two models with *w*_*i *_and  being the weights in the fixed- and random-effects model respectively. The ratio of information sizes needed in the two models may be calculated as:(2.3)

under the assumption that *μ*_F _= *μ*_R _it follows that:(2.4)

or(2.5)

the relationship between the *IS *(*N*_*R*_) for a random-effects model and the *SS *(*N*_*F*_) for a fixed-effect model is therefore multiplicative by an adjustment factor *A*_*RF*_(2.6)

Let *τ*^2 ^denote the between-trial variance, *k *the number of trials, and  the 'typical' moment-based sampling error within the trials according to Higgins and Thompson [10], then:(2.7)

and combining 2.6 and 2.7:

This yields the intuitive interpretation that the required *IS *in a random-effects model is a monotone increasing function of the degree of heterogeneity.

### 2.2 Limitations of a moment-based 'sampling error' in the definition of heterogeneity, I^2^

Higgins and Thompson [10] analysed candidate measures of intertrial variability and decided on the inconsistency factor *I*^2 ^under the assumption that all weights *w*_i _were approximately equal, that is,  for all *k *trials, that is: ∀*i *∈ {1,..., *k*} is . However, this assumption may not be met in many meta-analyses. In the attempt to generalise the use of *I*^2 ^to the situation with trial weights being unequal a 'typical' sampling error *σ*^2 ^of the included trials is assumed [10]. To use a 'typical' sampling error *σ*^2 ^may not be appropriate in some meta-analyses as weight percentages of the trials easily range from 0.5% to 50% or wider without a known distribution (Table 1 and Table 2). The attempt to estimate the trials' 'typical' sampling error as a moment-based sampling error [10] may be misleading as it attributes less emphasis to the trials with a very high number of participants and events. *I*^2 ^is interpreted as  and intends to measure the percentage of total meta-analysis variability explained by between-trial variation. In this vein,*I*^2 ^has been interpreted as the between trial variance relative to the sum of the between-trial variance and a 'typical' moment-based sampling error or as it has been phrased: "the between-trial variance rather than the sampling error"[10]. *I*^2 ^achieves some of the desired properties to characterise between-trial variability. However, the concept of a 'typical' sampling error is not relevant if it provides a misleading estimate, seriously distorting the *I*^2^calculation or interpretation. If  overestimates the sampling error then *I*^2 ^will be underestimated and vice versa. In such instances it may in fact be wise to abandon the concept of a 'typical' sampling error.

**Table 1:** Meta-analyses examples

Meta-analysis	Title	Intervention	Outcome measure	Number of trials	Number of participants
Afshari *and others* 2007 [16]	Antithrombin III for critically ill patients	Antithrombin III	Mortality	20	3,458
Al-Inany *and others*, 2006 [17]	Cycle cancellations due to poor ovarian response	Gonadotropin releasing hormone for assisted reproductive therapy	Number of cycle cancellations	13	2,543
Soll *and others*, 1997 [18]	Prophylactic surfactant to prevent morbidity and mortality in preterm infants.	Surfactant	Mortality or pneumothorax	8	988
Wetterslev and Juhl, 2006 [19]	Effect of perioperative *β*-blockade on non-fatal perioperative AMI	Perioperative *β*-blockers for non-cardiac surgery	Perioperative myocardial infarction within 30 days of operation	11	2,211
Bury and Tudehope, 2000 [20]	Effect of antibiotics on necrotizing enterocolitis in newborn	Enteral antibiotics in newborn	Necrotizing enterocolitis	5	458
Li *and others*, 2007 [21]	Intravenous magnesium for acute myocardial infarction	Magnesium	Mortality	23	72,472
Meyhoff *and others*, 2008 [22]	Perioperative ventilation with 80% versus 30% oxygen during intestinal surgery	Perioperative ventilation with 80% oxygen	Wound infection within 15 days of surgery	4	989

**Table 2:** Derived data from meta-analyses examples

Meta-analysis	Range of weights *w*_*i *_(% weights) in the fixed-effect model	Inconsistency (*I*^2^)%	Diversity (*D*^2^)%	(*D*^2 ^- *I*^2^)%	A priori relative risk reduction % (*RRR*)	Unadjusted information size (*SS*)	Heterogeneity-adjusted information size (*HIS*)	Diversity-adjusted information size (*DIS*)
Afshari *and others* 2007 [16]	0.2-281 (0.04-80%)	0.0	0.0	0.0	10	3,317	3,317	3,317
Al-Inany *and others*, 2006 [17]	0.2-3.9 (1-18%)	7.2	13.9	6.7	25	3,516	3,789	4,083
Soll *and others*, 1997 [18]	3.6-22.2 (6.2-38.1%)	22.9	37.3	14.3	60	193	250	307
Wetterslev and Juhl, 2006 [19]	0.3-10.4 (1-42%)	13.4	40.5	27.1	20	8,421	9,726	14,164
Bury and Tudehope, 2000 [20]	1.5 - 9.6 (7-38%)	40.2	57.7	17.5	45	440	736	1,039
Li *and others*, 2007 [21]	0.24-565.1 (0.02-42%)	61.9	89.9	28.0	10	31,094	81,466	306,276
Meyhoff *and others*, 2008 [22]	1.3-15.2 (4-47%)	74.2	79.4	5.2	30	1,699	6,581	8,239

If the focus is shifted towards a sufficient *IS *estimation, then adjusting factors based on *I*^2 ^calculated from a moment-based sampling error may be insufficient. We therefore suggest to consider an alternative adjusting factor to obtain an adequate estimation of the required *IS*.

### 2.3 Defining and implementing a measure of diversity

Assume we are interested in showing or rejecting a significant intervention effect, *μ*, regardless of the choice of meta-analysis model (fixed or random). That is, assume *μ *= *μ*_F _= *μ*_R_. We then define diversity (*D*^2^) as the quantity compelled to satisfy the following equation:(2.8)

Solving the equation with respect to *D*^2 ^we get the definition of *D*^2 ^explicitly:(2.9)

As long as we do not know what the difference between *I*^2 ^and *D*^2 ^covers, knowing now from 2.9 that *D*^2 ^reflects the total relative variance expansion changing from a fixed-effect into a random-effects model meta-analysis, we find it wise to denote *D*^2 ^diversity instead of just another calculation of heterogeneity. *A*_*RF *_will be an adjustment of *N*_*F *_to *N*_*R *_taking into account the total variance expansion changing from a fixed-effect into a random-effects model. Hereby, *D*^2 ^expresses the relative variance reduction when the model of meta-analysis is changed from a random-effects model into a fixed-effect model. *D*^2 ^is the percentage of change in variance when the model is changed. *D*^2 ^becomes exactly the proportion that the between-trial variance component (*τ*^2 ^= *k*·(*V*_*R *_- *V*_*F*_)) constitutes of the sum of variances () in the variance component model if and only if  (a sampling error originating from diversity or the required information size) is defined as:(2.10)

Diversity can then be expressed as:(2.11)

This way, *D*^2 ^in a meta-analysis may become a central measure of the between-trial variability relative to the sum of the between-trial variability with an estimate of the sampling error basically originating from the required information size.

As such, *D*^2 ^is able to quantify the relative model variance change from a random-effects into a fixed-effect model. More importantly *D*^2^, in contrast to *I*^2^, is not based on underlying assumptions of a 'typical' sampling error that are violated in most meta-analyses. *D*^2 ^is the percentage of the total variance (the sum of between trial variance and sampling error), in a random-effects model, contributed by the between trial variance.

### 2.4 Simulating meta-analyses

In our simulations, we considered meta-analyses with *k *= *6 *and *k *= *20 *trials. For each *k*, we considered the four combinations from two different average control event proportions, (*PC*) of 10% and 30%, and two true values of the overall effect in terms of odds ratios of 1 and 0.7. The above values were selected aiming to cover different plausible meta-analytic scenarios. In total, these values make up for 8 simulation scenarios.

For each combination of the above mentioned variables we generated data for *k *2×2 tables. For all *k *trials, within group sample sizes were determined by sampling an integer between 20 and 500 participants. Group sizes were equal in each simulated trial. We drew the trial specific control group event rate, *PC*_*i*_, from a uniform distribution, *PC*_*i*_~U(*PC*-0.15, *PC*+0.15). We drew the number of observed events in the control group from a binomial distribution *e*_*iC *_*~bin*(*n*_*i*_, *PC*_*i*_). For each meta-analysis scenario we varied the degree of heterogeneity by sampling the between-rial standard deviation, *τ *(not the between-trial variance *τ*^2^), from a uniform distribution, *τ ~U(10*^-10^, *SQRT(0.60))*. We simulated the underlying true trial intervention effects, as log odds ratio *ln(OR*_*i*_)~*N*(*OR, τ *^2^), where *OR *is the true intervention effect expressed as an odds ratio. We drew the observed number of events in the intervention group from a binomial distribution *e*_*iE*_*~bin*(*n*_*i*_,*PE*_*i*_), where *PE*_*i *_= *PC*_*i*_*exp(ln(OR*_*i*_))/(1 - *PC*_*i *_+ *PC*_*i*_*exp(ln(OR*_*i*_)))

For all meta-analysis scenarios we simulated 10,000 meta-analyses and for each of these we calculated the  and the . For each scenario we plotted *D*^2 ^against *I*^2 ^and incorporated the line of unity in the scatter-plot.

### 2.5 Selection of meta-analyses examples

We selected traditional random-effects meta-analyses to cover a range of inconsistency *I*^2 ^from 0% to 100% and to come from a wide range of medical research fields.

## Results

### 3.1 The relationship between diversity, D^2^, and heterogeneity, I^2^

We want to show that *D*^2 ^≥ *I*^2 ^for all meta-analyses. This is true if and only if:(3.1)

According to a special case of the Chebyshev's inequality [13] we arrange the weights so *w*_1 _≥ *w*_2 _≥ ...... *w*_*k*_., for any *k *≥ 0 we then get that:

and hence:(3.2)

and therefore(3.3)

and subsequently:(3.4)

We remember that Takouche et al. [14] proposed an estimate of a 'typical' sampling error , which yields the following relationship between  and :(3.5)

So it follows from (3.5) that  for all meta-analyses. Furthermore if we apply Chebyshev's inequality [13] arranging the weights  and at the same time *w*_1 _≥ *w*_2 _≥ ...... *w*_*k *_then:

 and as the random-effects weights are  and the fixed-effect weights are  we get:

and hence:

which leads to:

and subsequently:

and since:

it follows that:(3.6)

and therefore:

Remembering the definitions of  and  lead to:(3.7)

and it appears from (3.7) that  for all meta-analyses. As we have already shown in (3.5) that  it becomes clear that  in all meta-analyses. As  and with  it follows that:(3.8)

and, finally, *D*^2 ^≥ *T*^2 ^≥ *I*^2 ^in all meta-analyses.

### 3.2 Some useful properties of D^2^

Higgins and Thompson [10] specified three criteria that should be met by any quantification of variability between trials included in a meta-analysis: 1) the quantity should be a monotonically increasing function of the between-study variance, *τ*^2^; 2) the quantity should be scale invariant; and 3) the quantity should be independent of the number of included trials. It is easily verified that the *D*^2 ^fulfils the first two of these criteria. The third criterion may not be fulfilled, even by *I*^2 ^(simulations by K Thorlund, personal communication). However, *D*^2 ^becomes independent of the numbers of trials included in the meta-analysis, to the same degree as , because *D*^2 ^is a transformation of  fulfilling the criterion according to Higgins and Thompson [10]. Furthermore, it is easy to show that:(3.9)

demonstrating that the percentage of increase in variance when the model of meta-analysis is changed from a fixed-effect model into a random-effects model can, of course, also be expressed in terms of diversity.

It is equally clear that *D*^2 ^is always ≥ 0 as well as being < 1. *D*^2 ^is a fraction between 0 and 100% because:(3.10)

as (1 + *w*_i_·*τ*^2^) ≥ 1 for all *i *and for all estimators of *τ*^2 ^including the DerSimonian-Laird estimator [15] with  being at least greater than or equal to 0.

Furthermore, *D*^2 ^= *I*^2 ^when and only when all the weights *w*_i _in the fixed-effect model are equal. *D*^2^is approximately equal to *I*^2 ^if:(3.11)

Furthermore, *D*^2 ^= 0 when and only when *I*^2 ^= 0 because *I*^2 ^= 0 when and only when *τ*^2 ^= 0 the latter making  = 1 and hence .(3.12)

### 3.3 Simulations of meta-analyses

**Figure 1:**
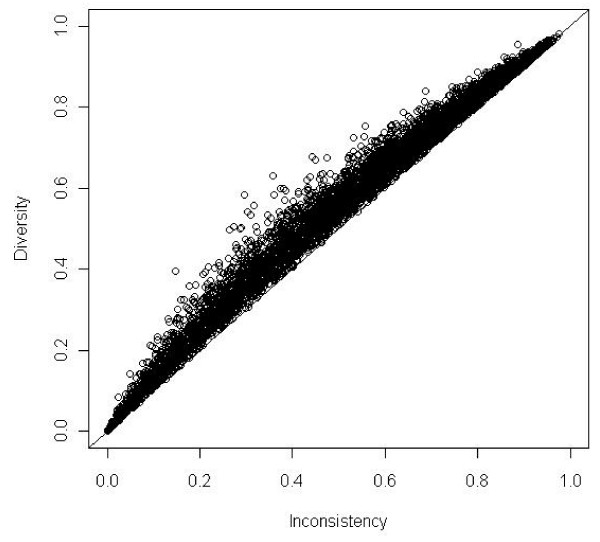
**Diversity (*D*^2^) compared to inconsistency (*I*^2^) in 10,000 simulations of meta-analyses with number of trials included *k *= 6**. Odds ratio = 1.00 and proportion of events in control group *PC *= 0.30. Meta-analyses depicted as open circles. *D*^2 ^nears asymptotically to *I*^2 ^when heterogeneity nears 0% or 100%. Line of unity, *D*^2 ^= *I*^2 ^black line.

**Figure 2:**
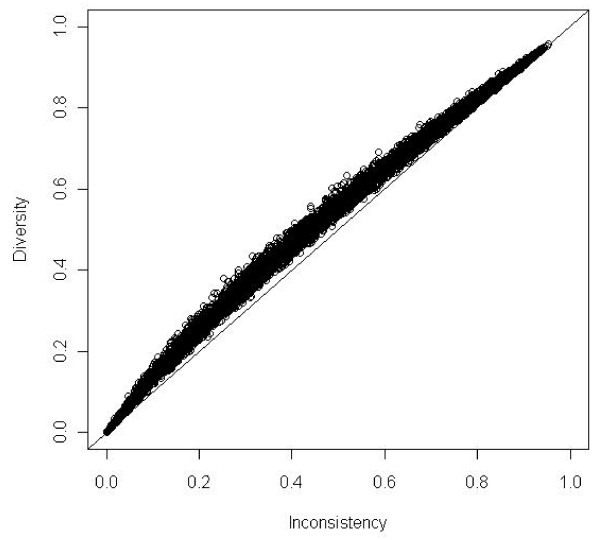
**Diversity (*D*^2^) compared to inconsistency (*I*^2^) in 10,000 simulations of meta-analyses with number of trials included *k *= 20**. Odds ratio = 0.70 and proportion of events in control group *PC *= 0.30. Meta-analyses depicted as open circles. *D*^2 ^nears asymptotically to *I*^2 ^when heterogeneity nears 0% or 100%. Line of unity, *D*^2 ^= *I*^2 ^black line.

We performed 8 simulation scenarios showing that *D*^2 ^always exceeds *I*^2 ^despite any assumptions. Meta-analyses with all weights being equal corresponding to *D*^2 ^= *I*^2 ^were rare. The pattern of data showed a greater degree of scatter in the scenarios where k = 6. The results of the simulations of 10 000 meta-analyses according to the combinations of *OR *= 0.70, *OR *= 1.00 and *PC *= 30% with 6 and 20 trials, respectively, are presented in figure 1 and figure 2. As seen *D*^2 ^exceeds *I*^2 ^for all the simulated meta-analyses independent of the chosen *OR *and number of trials in the meta-analyses.

### 3.4 Examples

We used the expression of *D*^2 ^to calculate this quantity in seven traditional random-effects meta-analyses [16-22] listed in Table 1. These meta-analyses cover a range of inconsistency, *I*^2^, from 0% to 74.2% and come from different medical research fields: intensive care [16], assisted reproductive technology [17], perioperative medicine [19,22], neonatology [18,20], and cardiology [21]. The results of the calculations of *I*^2^,*D*^2^, inconsistency-adjusted information size *HIS *(), and diversity-adjusted information size *DIS *() from these meta-analyses are shown in Table 2. The range of the calculated unadjusted *SS *range from 440 to 31,094 participants.

**Figure 3:**
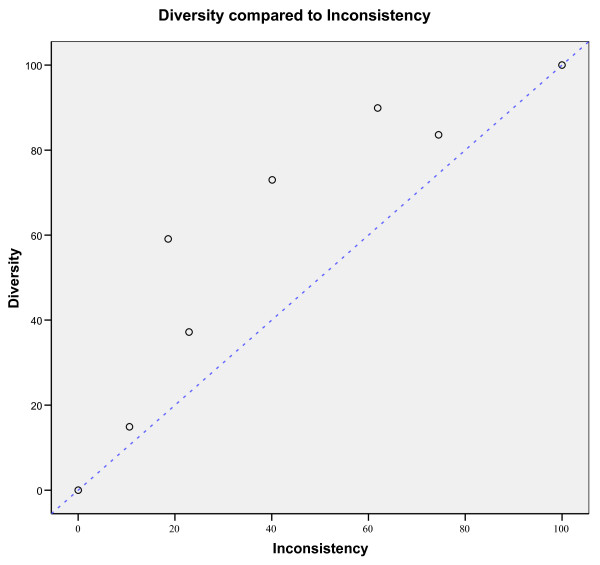
**Diversity (*D*^2^) in % compared to inconsistency (*I*^2^) in % in seven meta-analyses (see Table 1) depicted as open circles**. The open circles indicate that *D*^2 ^is always equal to or larger than *I*^2^. 100% heterogeneity is impossible and the upper right point is just to illustrate that *D*^2 ^nears asymptotically to *I*^2 ^when heterogeneity nears 100%. Line of unity, *D*^2 ^= *I*^2 ^is the dotted blue line.

Figure 3 shows the relationship between *D*^2^, *I*^2^, and unity. All the meta-analyses examples are shown as open circles above the line of unity as *D*^2 ^≥ *I*^2^. The difference (*D*^2 ^- *I*^2^) increases with heterogeneity until a certain point, after which the difference again regresses to 0.

## Discussion

Using a mathematical derivation, meta-analyses simulations, and examples of meta-analyses we derive a concept of diversity, *D*^2^. *D*^2 ^may be used for adjustment of the required information size in any random-effects model meta-analysis once the between trial variance is estimated. Focusing on the required information size estimation in a random-effects meta-analysis, *D*^2 ^seems less biased compared to *I*^2^. The *D*^2 ^is directly constructed to fulfil the requirements of the information size calculation and is subsequently independent of any 'typical' *a priori *sampling error estimate, whereas the *I*^2 ^is influenced by an *a priori *'typical' sampling error estimate. We therefore find that it is possible and appropriate taking *D*^2 ^into consideration to calculate the required *IS *in meta-analyses as *DIS*.

*DIS *has several advantages. It measures the required *IS *needed to preserve the anticipated risk of type I and type II errors in a random-effects model meta-analysis. *DIS *considers total variance change when the model shifts from a fixed-effect into a random-effects model. *DIS *is a model dependent and derived estimate of the required *IS*. The adjustment is dependent only on the anticipated intervention effect and on the model used to incorporate the between-trial variance estimate . *D*^2 ^applies to random-effects models other than that proposed by DerSimonian-Laird [16] as long as the between-trial estimator, , is specified. The adjustment of *IS *does not depend on the level of type I and II errors, as (*Z*_1-*α*/2 _+ *Z*_1-*β*_)^2 ^is levelled out during the derivation of the adjustment factor *A*_*RF *_(see equation 2.1, 2.2, and 2.5). The relationship *D*^2 ^≥ *I*^2 ^in all the simulations and in all the examples (shown as points above the line of unity in figure 1, 2, and 3) are in accordance with the properties of *D*^2 ^compared to *I*^2 ^derived in section 3.1.

There are limitations of *DIS*. Like *HIS *the use of *DIS *cannot compensate for systematic bias such as selection bias, allocation bias, reporting bias, collateral intervention bias, and time lag bias [5,23-28]. Furthermore, *DIS *is always greater than or equal to *HIS*, which may emphasise that caution is needed when interpreting meta-analysis before the required *DIS *has been reached [2-8].

The calculation of *HIS *and *DIS *may seem to contrast the *SS *calculation in a single trial where no adjustment for heterogeneity or diversity is performed. However, Fedorov and Jones [29] advocated the necessity of adjusting *SS *for heterogeneity arising from different accrual numbers among centres in a multi-centre trial in order to avoid the trial being underpowered. If such an adjustment seems fair for a single trial, it also appears appropriate for a meta-analysis of several trials. As an example, we calculated the *DIS *to 14,164 participants for a meta-analysis of the effect on mortality of perioperative beta-blockade in patients for non-cardiac surgery (Table 2). This may explain why a recent meta-analysis of seven randomised trials with low-risk of bias including 11,862 participants indicates, but still does not convincingly show, firm evidence for harm [30]. The actual accrual of 11,862 participants is beyond the *HIS *of 9,726 participants, but below the *DIS *of 14,164 participants, and the meta-analysis [30] may still be inconclusive. This suggest that *HIS *is not a sufficiently adjusted meta-analytic information size. Furthermore, the example demonstrates the important question of the stability of *I*^2 ^and *D*^2 ^beyond a certain number of trials in a meta-analysis as *I*^2 ^was 13.4% in the meta-analysis after 2,211 participants [19] and has now doubled to *I*^2 ^= 27.0% after 11,862 accrued participants in the meta-analysis of seven trials with low-risk of bias [30]. The assumption of *I*^2 ^and *D*^2 ^becoming stable after five trials is probably wrong and illustrates the moving target concept, which we have to face doing cumulative meta-analysis as evidence accumulates. Although a moving target may cause conceptual problems, a moving target may be better than no target at all.

The assumption that the *IS *required for a reliable and conclusive fixed-effect meta-analysis should be as large as the *SS *of a single well-powered randomised clinical trial to detect or reject an anticipated intervention effect [2-4] may not be necessary in some instances. The statistical information (*SINF*) required in a meta-analysis could ultimately be expressed as [31], with *δ *being the effect size. As *SINF *is the reciprocal of the variance in the meta-analysis, say , it follows that in meta-analyses with , the amount of information may eventually suffice to detect, or reject, an effect size of *δ*, without yet having reached *HIS *or *DIS*. This criterion, however, is not a simple one and may only be fulfilled occasionally. Furthermore, it seems impossible to forecast or even to get an idea of the magnitude of  in the beginning of a series of trials as well as along the course of trials being performed.

*D*^2 ^offers a number of useful properties compared to *I*^2^. In contrast to *I*^2^, *D*^2 ^reflects the relative variance expansion due to the between trial variance estimate  without assuming an estimate of a 'typical' sampling error *σ*^2^. *D*^2 ^is reduced when the estimate  is reduced, even for the same set of trials. In case diversity is larger than inconsistency this may be an indication that total variability among trials in the meta-analysis is even greater than suggested by *I*^2^. *I*^2 ^is intrinsically influenced by a potentially overestimated sampling error (), thereby underestimating  and inherently placing less weight on large trials with many events. On the other hand a 'typical' sampling error originating from the required information size, , could be deduced from the *D*^2^. We would, however, advise great cautiousness in such an attempt. The difference (*D*^2 ^- *I*^2^) reflects the difference of the moment-based and the information size-based 'typical' sampling error estimate. The calculation of diversity and (*D*^2 ^- *I*^2^) may serve as supplementary tools to the assessment of variability in a meta-analysis. *D*^2 ^is a transformation of the variance ratio of the variances from the random-effects model and the fixed-effect model. This variance ratio was a candidate for the quantification of heterogeneity [10].

*D*^2 ^may vary within the same set of trials when different between trial variance estimators  are used in the corresponding random-effects model. On the contrary, *I*^2 ^is intimately linked to the specific between trial variance estimator in the DerSimonian-Laird random-effects model as *I*^2 ^by definition is [10] and *Q *is used to estimate a moment-based between trial variance [15]. The interpretation of heterogeneity  is obviously dependent on the variance estimator  as well. An estimate of *τ*^2 ^is a prerequisite for any random-effects model and the actual estimated value, together with the way  is incorporated into the model, actually constitutes the model [32]. Therefore, a quantification of the between-trial variability rather than sampling error which is independent of the specific random-effects model is impossible, as it is constituted by the between trial variance estimator [32]. *D*^2 ^adapt automatically to different between trial variance estimators [32] while *I*^2 ^is linked to the estimator from the DerSimonian-Laird random-effects model.

*D*^2 ^may have some limitations too. The derivation of *D*^2 ^depends on the assumption that the point estimate of the intervention effect in the fixed-effect model and the point estimate of the intervention effect in the random-effects model are approximately equal. Meta-analyses with considerable difference of the point estimate in the fixed-effect model and the point estimate in the random-effects model represent specific problems. Probably more information is needed when *μ*_*F *_>> *μ*_*R *_since the formula  yields higher values for *N*_*R *_under the assumption of a constant variance ratio. On the other hand less information may be needed when *μ*_*F*_<<*μ*_*R *_since the formula  then yields lower values for *N*_*R *_under the assumption of a constant variance ratio. However, examples with considerable differences of the point estimates in a fixed- and random-effects model presumably represent meta-analyses of interventions with considerable between trial variance due to small trial bias. The meta-analysis of the effect of magnesium in patients with myocardial infarction is such an example [21] where one large trial totally dominate the result in the fixed-effect model but are unduly down-weighted in the random-effects model. Care should be taken to interpret the random-effects model despite any calculated information size in such a situation. Further, to foresee *a priori *the size of the difference between *μ*_*F *_and *μ*_*R *_seems impossible and the calculation may then degenerate exclusively to a post hoc analysis.

Second, *D*^2^, though potentially unbiased with respect to information size calculations, could come with a greater variance than *I*^2 ^when both are calculated in the same set of meta-analyses. This latter situation presents a potentially unfavourable 'bias-variance-trade off' but an estimate of its magnitude will have to await simulation studies addressing the issue.

It may seem an advantage that *I*^2 ^is always reported in meta-analysis and therefore readily available to adjust the expected information size. On the other hand  is also calculable for meta-analysis of ratio measures (e.g, RR or OR), width_F _and width_R _refers to the widths of the confidence intervals for the logarithmic transformed measures in the fixed-effect and the random-effects models, respectively.

Last but not least the decision to pool intervention effect estimates in meta-analysis should be the clinical relevance of any inconsistency or diversity present. The between trial variance,*τ*^2^, rather than *I*^2 ^or *D*^2^, may be the appropriate measure for this purpose [33-35].

The estimation of a required *IS *for a meta-analysis to detect or reject an anticipated intervention effect on a binary outcome measure should be considered based on reasonable assumptions. Accordingly, it may not be wise to assume absence of heterogeneity in a meta-analysis unless the intervention effect is anticipated to be zero [36,37]. On the contrary it may be wise to anticipate moderate to substantial heterogeneity (e.g., more than 50%) in an *a priori *adjustment of the required *IS *[37]. The concept of diversity points to the fact that an adjustment based on the experience with inconsistency would result in underestimated heterogeneity and hence an underestimated required *IS *[37]. Alternatively for a future updated meta-analysis to become conclusive we may apply the actual estimated heterogeneity of the available trials in a meta-analysis as the best we have for the adjustment of the required *IS*. *D*^2 ^seems more capable than *I*^2 ^in obtaining such an adequate adjustment.

## Conclusion

A quantity to characterise the proportion of between trial variation in any meta-analysis relative to the total model variance of the included trials is needed. Diversity, *D*^2^, may be such a quantity. *D*^2 ^describes the relative model variance reduction changing from a random-effects model into a fixed-effect model. Diversity may be described as the proportion of the total variance in a random-effects model contributed by the between trial variation despite the chosen between trial variance estimator. Furthermore, *D*^2 ^can adequately adjust the required information size in any random-effects meta-analysis irrespective the meta-analytic model.

## List of abbreviations

*α*: Risk of type 1 error; *β*: Risk of type 2 error; *A*_*RF*_: Adjustment factor of information size changing from a fixed-effect to a random-effects model; ∀: For any...; *Q*: Cochran's *Q*; *D*^2^: Diversity; *DIS*: Diversity adjusted information size (); *HIS*: Heterogeneity adjusted information size (); *I*^2^: Inconsistency factor; *K*: Number of trials in a meta-analysis; *N*_R_: Required number of participants in a random-effects meta-analysis; *N*_F_: Required number of participants in a fixed-effect meta-analysis; *IS*: Required number of participants in a meta-analysis; *μ*_F_: Estimate of the intervention effect in a fixed-effect meta-analysis; *μ*_R_: Estimate of the intervention effect in a random-effects meta-analysis; *OR*: Odds ratio; *PC*: Control event rate; *RRR*: Relative risk reduction; *SS*: Sample size in a single randomised clinical trial; : Estimate of a typical sampling error considering diversity; : Estimate of a typical moment-based sampling error; : Mean of estimates of sampling errors in a meta-analysis; *τ*^2^: Estimator of the variance of between trial intervention effect estimates; : Estimate of the variance of between trial intervention effect estimates; : DerSimonian-Laird estimate of the variance of between trial intervention effect estimates; *V*_*F*_: The variance in a fixed-effect meta-analysis; *V*_*R*_: The variance in a random-effects meta-analysis; *Z*_1-*α*/2_: Fractile for 1-*α*/2; *Z*_1-*β*_: Fractile for 1-*β*.

## Conflicts of interests

The authors declare that they have no competing interests.

## Authors' contributions

JW and KT conceived the idea of an information size adjustment factor and JW devised the concept of diversity, made the mathematical derivations and calculated the examples. KT sat up the simulation program and KT and JW performed the simulations. JW, KT, and CG drafted the first manuscript. KT, JB and CG suggested revisions implemented by JW.

## Authors information

JW is an anaesthesiologist and a trialist working with meta-analysis and trial sequential analysis at the Copenhagen Trial Unit having special interests in perioperative medicine.

KT is a biostatistician working with meta-analysis and trial sequential analysis at the Copenhagen Trial Unit.

JB is an intern working in paediatrics with meta-analysis and trial sequential analysis.

CG is head of the Copenhagen Trial Unit, Editor-In-Chief of the Cochrane Hepato-Biliary Group, a trialist, and an associate professor at Copenhagen University.

## Pre-publication history

The pre-publication history for this paper can be accessed here:

http://www.biomedcentral.com/1471-2288/9/86/prepub
